# Peculiarities of the Pulse

**Published:** 1892-03

**Authors:** 


### PECULIARITIES OF THE PULSE.

Being able to "take" one's own pulse is a doubtful accomplishment, because the heart has some peculiarities, the importance of which are sure to be overestimated except by physicians, and much uneasiness occasioned in consequence. Irregularity of the pulse is natural to no small number of people without other signs of disease. It may also be simply a transient symptom, due to errors of habit or other causes, which, disappearing, leave no trace behind them.

There are infinitely many influences that are capable of disturbing the heart's action, and so rendering the pulse irregular, that are yet not likely to be productive of disease of that vital organ, unless, of course, they persist for a long time. Among these are unusual activity, excitement, nervousness, excessive smoking of tobacco, intemperate use of coffee, tea or other stimulants, overeating, dyspepsia.

The most common irregularity produced by these is nervous palpitation, during an attack of which the pulse may range wide, and from thirty to one hundred beats per minute. This affection is a common symptom of organic disease of the heart, and yet it very frequently occurs independently of such disease, in which case, although it is quite certain to cause the victim great uneasiness, it never destroys life.

Intermittency is that form of irregularity in which the pulse appears to drop a beat occasionally. In some instances it occurs regularly, and two or three times per minute for several hours; sometimes, also, it is very irregular, and is noted a number of times within a few seconds, and not again for a minute or more. This peculiarity, also, generally causes much uneasiness; yet, while it may be a very serious symptom, and associated with grave and incurable disease of the heart, it often signifies merely a functional disturbance which is in no wise dangerous.

When not caused by organic disease of the heart, intermittency is usually occasioned by nervousness, produced by tobacco, coffee, tea, loss of sleep, mental overwork, grief, anxiety, or other like disturbing influences. It is also quite common in dyspepsia, in which affection it is most often noted when the patient is lying down. Remove these, and the pulse in time again becomes regular.

Persons often consult their physicians because of distinct variations in their pulse, which beats naturally for a number of seconds and then quickens and takes on greater force, but after a short interval returns to the normal. This irregularity, while it occurs in heart disease, may yet be induced by the same disturbances that often give rise to intermittency.

Many other variations in the pulse are observed, but sufficient for the purpose has been considered. It is evident that to the layman the pulse is of but little value as a diagnostic sign. Occasional and fleeting changes in it, while the health seems good, should not occasion uneasiness. If irregularities occur often, or are quite persistent, or if any are noted while the health is impaired, then the attention of a physician should be called to them.

